# The role of face masks within in-patient psychotherapy: Results of a survey among inpatients and healthcare professionals

**DOI:** 10.3389/fnins.2022.1030397

**Published:** 2022-12-08

**Authors:** Rebecca Erschens, Sophia Helen Adam, Chiara Weisshap, Katrin Elisabeth Giel, Hannah Wallis, Anne Herrmann-Werner, Teresa Festl-Wietek, Nazar Mazurak, Stephan Zipfel, Florian Junne

**Affiliations:** ^1^Department of Psychosomatic Medicine and Psychotherapy, Internal Medicine, University Medical Hospital Tübingen, Tübingen, Germany; ^2^Department of Psychosomatic Medicine and Psychotherapy, Otto-von-Guericke-University Magdeburg, Magdeburg, Germany; ^3^Tübingen Institute for Medical Education, University of Tübingen, Tübingen, Germany; ^4^Centre of Excellence for Eating Disorders Tüebingen (KOMET), Tübingen, Germany

**Keywords:** face masks, inpatient, psychotherapy, healthcare professionals, COVID-19, in-patient psychotherapy

## Abstract

**Introduction:**

Face-to-face medical and psychotherapeutic treatments during the Corona pandemic often involve patients and health care providers wearing face masks. We performed a pilot survey assessing the subjective experience of wearing face masks during psychotherapy sessions regarding (i) feasibility, (ii) psychotherapeutic treatment and (iii) communication, emotion and working alliance in patients and healthcare professionals.

**Methods:**

A total of *n* = 62 inpatients (RR = 95.4%) and *n* = 33 healthcare professionals (RR = 86.8%) at an academic department of Psychosomatic Medicine and Psychotherapy participated in this survey anonymously. The items of the questionnaire were created by the interprofessional expert team and were based on existing instruments: (i) the Therapeutic Relationship Questionnaire and (ii) the German translation of Yalom’s Questionnaire on Experiencing in Group Psychotherapy.

**Results:**

The majority of patients rate their psychotherapy as highly profitable despite the mask. In individual therapy, face masks seem to have a rather low impact on subjective experience of psychotherapy and the relationship to the psychotherapist. Most patients reported using alternative facial expressions and expressions. In the interactional group therapy, masks were rather hindering. On the healthcare professional side, there were more frequent negative associations of face masks in relation to (i) experiencing connectedness with colleagues, (ii) forming relationships, and (iii) therapeutic treatment.

**Discussion:**

Information should be given to patients about the possible effects of face masks on the recognition of emotions, possible misinterpretations and compensation possibilities through alternative stimuli (e.g., eye area) and they should be encouraged to ask for further information. Especially in group therapy, with patients from other cultural backgrounds and in cases of need for help (e.g., hearing impairment) or complex disorders, appropriate non-verbal gestures and body language should be used to match the intended emotional expression.

## Introduction

The global COVID-19 pandemic challenges the healthcare system on multiple fronts and has profound effects on the daily lives of people worldwide (Brooks et al., 2020; Mazza et al., 2020; Pierce et al., 2020). Meta-analyses (Vindegaard and Benros, 2020; Busch et al., 2021; Danet, 2021; Dragioti et al., 2022) show a significant increase in mental distress among healthcare professionals (Mulfinger et al., 2020; Salazar de Pablo et al., 2020; Jones et al., 2021) in addition to the significantly increased prevalence of mental distress in general (Xiong et al., 2020; Santomauro et al., 2021). Alongside the medical care of SARS-CoV-2 patients, the continued treatment of all other patients must also be ensured. The pandemic has also led to changes in the provision of psychotherapeutic services: while psychotherapy has partly been conducted using telephone or videoconferencing throughout the pandemic, face-to-face psychotherapy continued to be offered, especially for severely ill patients in day-patient and inpatient settings (Zipfel et al., 2020).

Among many benefits of telemedicine and telepsychotherapy (Poletti et al., 2021; Lin et al., 2022) challenges remain, such as a “lack of control over the patient’s environment,” reduced privacy and confidentiality, and possibly limited assessment of treatment progress and difficulties in establishing a therapeutic relationship without face-to-face contact (Cataldo et al., 2021; Mitzkovitz et al., 2022).

Face-to-face psychotherapy has changed in many ways: smaller group sizes, a distance of at least 1.5–2 m, and refrain from physical contact, healthcare professionals, and patients wear face masks in multiple medical and therapeutic contexts. Face masks cover/conceal important facial features of non-verbal communication, more specifically the lower part of the face. Between 65 and 90 per cent of human communication is non-verbal, with all communication containing a contextual and a relational aspect, with the latter determining the former (Foley and Gentile, 2010). In general, the contextual aspect has the task of conveying information. The relational aspect provides information about how the relationship is perceived by the receiver. In this context, non-verbal communication is conveyed through body posture, facial expressions, gestures, speech quality, and predispositions, among other things (e.g., Watzlawick, 1969; Foley and Gentile, 2010; Wieser and Brosch, 2012). In the upper half of the face, movements such as lifting and contracting the eyelids and raising and lowering the eyebrows occur, whereas in the lower half of the face (movements such as), pulling the corners of the lips, splitting the lips, and lifting the lips occur. Different emotions are predominantly handled by functional areas of the upper or the lower face. Anger and sadness are more likely to be addressed by lower functional areas, while both halves of the face are relevant for fear and surprise. The loss of information from the lower face through the mask thus increases the ambiguity of a message (Carbon, 2020; Ekman and Rosenberg, 2020; Pavlova and Sokolov, 2021).

Drawing from these findings we were interested in potential side-effects of wearing face-masks on the therapeutic relationship. The therapeutic alliance is a key factor for positive treatment outcomes (Flückiger et al., 2018). On the one hand, masks carry the risk of misrecognizing facial expressions, especially if the expression does not match the corresponding body language. This is particularly relevant for patient groups for whom emotion recognition is a problem, or who are more susceptible to emotion recognition bias (e.g., Mitzkovitz et al., 2022). Based on previous research on the perception of masks among individuals suffering from health anxiety (Cannito et al., 2020), an attentional bias toward virus-relevant stimuli (i.e., face masks) and thus interactionally disruptive effects on psychotherapy may be assumed. Similarly, face masks could create unfamiliar distance and impair the feeling of coherence (e.g., Wong et al., 2013). As recent research in the context of the COVID-19 pandemic has shown, there is also a striking influence on the trustworthiness of the interaction partner through the wearing of a mask (Biermann et al., 2021; Malik et al., 2021; Cannito et al., 2022; Marini et al., 2022). As proposed in the concept of epistemic trust (Fonagy and Allison, 2014), basal trust in a reference person as a secure source of information can be perceived as core element of a functioning, resilient therapeutic relationship. This relationship, or rather the successful therapeutic relationship building, may therefore be impaired by the wearing of a mask (Cannito et al., 2022).

However, face masks can create possible opportunities for increased abstinence on the part of the professional and increased problem activation on the part of the patient (Grawe, 1997). Recent research has shown that wearing a mask is not only obstructive. Marini et al. (2022) have shown that wearing a mask may mitigate positive and negative perception biases since visual information underlying trustworthiness is also available in masked faces (Marini et al., 2022). Both can be an opportunity and an overload depending on the current stress, resource activation, problem, and structural level.

The Aim of this study was to explore inpatients and healthcare professionals from a German tertiary hospital in a department of Psychosomatic Medicine and Psychotherapy concerning their subjective perceptions and experience of wearing face masks within psychotherapy during the first wave of COVID-19 outbreak in Germany in regard to three dimensions:

1. general feedback and feasibility;
2. specific psychotherapeutic treatment; and
3. communication, emotion, and relationships with others.

To the best of our knowledge, there has been no similar study on the above-mentioned relationships.

## Materials and methods

### Material and procedure

The study was conducted in a German tertiary hospital in a department of psychosomatic medicine and psychotherapy using a paper-and-pencil survey. The participant sample included inpatients and healthcare professionals, including psychotherapeutically trained nurses, physicians, psychotherapists, and specialty therapists. Between July and October 2020, *n* = 65 inpatients and *n* = 38 medical healthcare professionals were invited to participate in the survey. Therefore, we invited all patients who were hospitalized during this period, as well as all health professionals involved in therapeutic activities during this period, to participate in the study. Participants were informed about the purpose of the study, the study investigators, and the use of non-personal data by a study information sheet. The paper-pencil questionnaire was completed voluntarily and without any consequences for the participants. The questionnaire was handed out by a scientific research associate (CW), not integrated in psychotherapeutic treatment. Patients received the questionnaire within the first 2 weeks after admission. After completing the anonymous questionnaire, participants could drop the questionnaire into a locked box.

The questionnaire included *n* = 60 (patient version) vs. *n* = 92 (staff version) items concerning experiences and perceptions of wearing face masks in psychotherapy, both on patient and staff side. They were developed by an interprofessional team (psychologists, psychotherapists, clinical scientists, physicians, nurses, and special therapists) and were also inspired by existing validated instruments like the Therapeutic Relationship Questionnaire (Schulte, 1996) and the German translation of Yalom’s Questionnaire on Experiencing in Group Psychotherapy (Mander et al., 2016). The Questions were asked dichotomy [“Yes, face mask affects (complicates or encourage) this area/item” or “No, face mask does not affect this area/item”]. Furthermore, free text fields were provided for each domain. Table 1 shows 10 exemplary items for both groups. Please note that in addition to their subjective experience regarding the face masks, the healthcare professionals also gave a rating for the potential effects of face masks on patients. No person-specific information or information on personal data such as gender, age, type of diagnosis, or clinical parameters was obtained.

**TABLE 1:** Illustration of ten exemplary items for both groups.

Patient side	Health professional side
	
Face mask complicates/encourages… OR face mask does not affect…	Face mask complicates/encourages… OR face mask does not affect…
My communication with fellow patients.	My personal wellbeing negatively/positively.
Me feeling understood in my problems by my therapist.	My patients knowing what I expect from them.
My personal therapeutic work with the nursing team.	Talking (consciously) to patients in a more resource-oriented way (such as “auxiliary I,” more praise, appreciative language, more validation, clearer language).
Me feeling free to shape the course of therapy.	My patients confiding intimate things to me.
Me approaching the staff for help.	My patients coping well with difficult situations in the group therapy.
Me feeling safe and secure in the group.	The therapeutic work in music or art therapy.
Learning something from other patients in the group sessions (e.g., experiences, perspectives, strategies).	My patients feeling understood by me in their problems.
Addressing many things that are important for me in the individual therapy.	My patients developing positive feelings about their future in the group therapy.
Me feeling valued and/or understood by the other patients in the group therapy sessions.	A lot of uncertainty in the team.
Me feeling that the therapist pays enough attention to my feelings.	My patients feeling important and valuable in the group therapy/individual therapy.

The responsible ethics committee of the University Hospital and Medical Faculty of the University of Tübingen was informed (project number: 685/2002A). For completely anonymous data, consultation and approval on the collection, analysis, and publication by the ethics committee is not required.

### Analysis of data

Statistical analyses were carried out using IBM SPSS for Windows, version 27 (IBM Corp., Armonk, NY, USA). We calculated frequencies for agreeing and disagreeing with a subjectively perceived effect of the mask on each question. Given this data evaluation procedures, no power calculation (*a priori* or *posteriori*) was necessary. The free text responses addressed were analyzed with a thematic content analysis using Microsoft Excel as the coding software (in regard to Braun and Clarke, 2006). Codes and dimensions from the data set were identified, analyzed, and documented. During the content analysis, the reviewers (RE and CW) familiarized themselves with the data and developed codes. Following the search, exploration and specification of the themes, the results of the analysis were interpreted and integrated for both the questionnaire results and the free text fields (RE and SHA). The resulting dimensions were:

(i) general feedback and feasibility;
(ii) psychotherapeutic treatment; and
(iii) communication, emotion, and relationships (including team members, patient–to patient, and patient–healthcare professionals).

## Results

**FIGURE 1:**
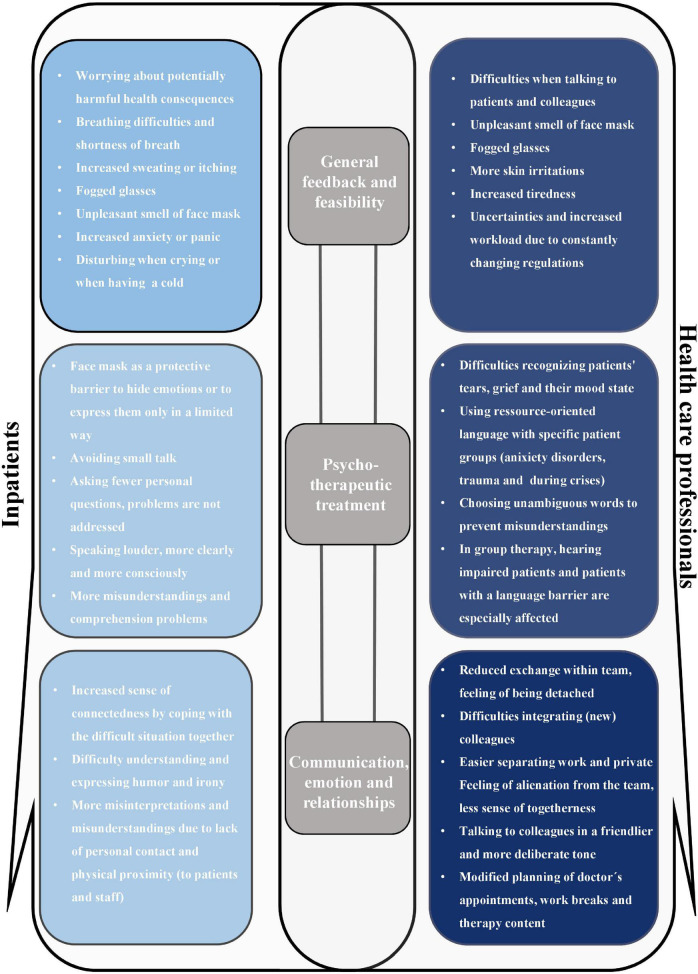
Visualization of the qualitative results in the three dimensions (i) General feedback and feasibility, (ii) Psychotherapeutic treatment, and (iii) Communication, emotion, and relationships with others by healthcare professionals **(right)** and patients **(left)**. For each dimension (i–iii), the most frequent statements of the free text fields were compiled.

A total of 62 inpatients (RR = 95.4%) and 33 healthcare professionals (RR = 86.8%) took part in this survey. Seven patients and five healthcare professionals had to be excluded from the further analysis due to incomplete (>20%) or ambiguous answers. A total of *n* = 55 patients (84.6%) and 28 healthcare professionals (73.7%) were included finally in the following analyses. Figure 1 illustrates the qualitative results within the three dimensions by healthcare professionals and patients.

### General feedback and feasibility

Over 95% of the respondents (patients and healthcare professionals) reported wearing face masks according to the ward rules. Overall, 68% (*n* = 19) of healthcare professionals reported difficulty wearing the face mask compared to *n* = 36 (65.5%) of patients. In total 62% of the healthcare professionals reported gradually getting used to wearing the mask, while only about 40% (*n* = 22) of the patients managed to do so.

Both groups were concerned that face masks would be detrimental to their health. They stated that face masks emitted unpleasant odors, that the masks led to increased breathing difficulties and panic attacks, and that there was increased sweating and feelings of anxiety and panic (on the part of the patients). On the practitioner’s side, it was emphasized that stair climbing was impaired under the mask. A total of 54% (*n* = 15) of the healthcare professionals stated that the face mask had a negative influence on their own wellbeing. A total of 74% (*n* = 27) of the healthcare professionals were able to recognize their colleagues “at first sight” even with the mask. On the patient side, *n* = 43 (79.9%) stated that they could recognize their fellow patients at first sight despite the face masks as well. The majority of patients, *n* = 45 (84.9%), stated that the face mask did not prevent them from approaching staff (or fellow patients) and asking for help.

### Psychotherapeutic treatment

**FIGURE 2:**
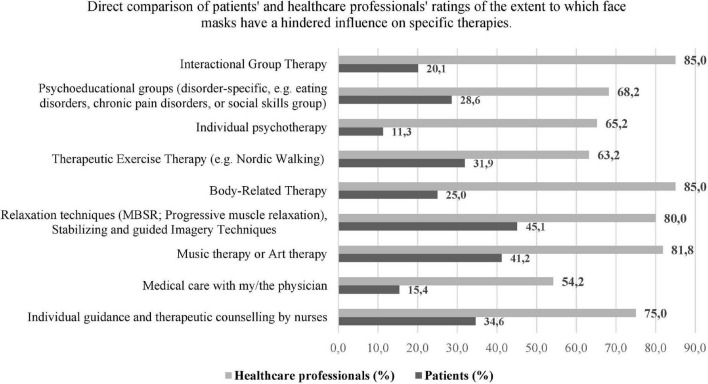
Illustration of the ratings, shown as percentages of frequency of patients (*n* = 55) and healthcare professionals (*n* = 28) interviewed regarding a disturbing effect by the face masks on various types of therapy. The percentages refer to the “applicable percentages” of the individual items.

Figure 2 provides information on the direct comparison of patients’ and healthcare professionals’ ratings of the extent to which face masks have a negative/hindered influence on specific therapies. The percentages refer to the “applicable percentages” of the individual items. The healthcare professionals’ individual ratings of the impact of facial masks are consistently more likely to be negative (range 54.2–85.0%) than the patients’ ratings (range 11.3–45.1%).

The interfering effect of the face mask varied depending on the respective type of therapy. Face masks were experienced as particularly problematic by patients in “Music therapy or Art therapy” (*n* = 21, 41.2%), “Relaxation therapy” (*n* = 23, 45.1%), and individual guidance and therapeutic counseling by nurses with *n* = 18, 34.6%. For healthcare professionals, the top three therapies in which facial expression was experienced as particularly interfering were “Interactional Group Therapy” and “Body-Related Therapy” with *n* = 17, 85.0% each, and “Music therapy or Art therapy” (*n* = 18, 81.8%). Regarding individual therapy, the difference becomes even more apparent. More than 65% of the healthcare professionals estimate a negative influence of face masks in individual psychotherapy. More precisely, 33% of the healthcare professionals thought that the face mask prevented patients from feeling understood by the therapist, or that they did not feel sufficiently listened to (28.6%), or that they are perceived as honest by the patients (29.2%). The majority of healthcare professionals, however, reported not consciously working in a more resource-oriented or less confrontational or exposed way (*n* = 23; 82%) when wearing the face mask. Methods such as “auxiliary I” (in case of structural ability) increased positive feedback, more appreciative language, more intensive validation, more unambiguous language were, however, more likely to be used with patients with complex disorders such as anxiety, post-traumatic stress disorders, major depression, and comorbid personality disorders, as well as in conflict situations.

Only 11.3% of the interviewed patients reported an interfering experience with the face mask in the individual therapy. In the further analysis (multiple answers were possible): from out of the seven of the 55 interviewed patients who stated a negative influence of the face mask, six patients stated that they felt less understood. Three patients stated that the face mask prevented them from working with the psychotherapist to achieve common goals/aims and five of the patients experienced an inhibition to address relevant topics in therapy. Furthermore, 80% of all interviewed patients stated that they felt understood by their psychotherapists despite the face mask, more than 90% of the patients perceived the psychotherapists as “honest.” A total of 84% even declared that they could trust and 93% stated that they felt well supported by their psychotherapists.

In total, 85% of the healthcare professionals stated that the face masks had an interfering effect on the interactional group therapy, while only 21% of the patients generally assessed it this way. In a detailed evaluation, 29% of these patients then stated that they had difficulties in communicating with the psychotherapists and with other patients in the group. More than 38% saw a barrier in showing their true feelings to the other group members. A total of 32% said that the face masks made them less aware of their effect on the other patients in the group and 34% admitted that the face mask made it more difficult to react to challenging situations in the group. The healthcare professionals stated in the free text fields that patients with impaired hearing and patients with language barriers in particular had increased problems in group therapy due to the face mask.

### Communication, emotion, and relationships

About 86% (*n* = 19) of the healthcare professionals stated that face masks changed the way they formed relationships with the patients. The majority of patients (84.9%), reported that the face mask did not prevent them from approaching staff (or fellow patients) and asking for help. A total of 78.8% of the patients (89% of the healthcare professionals, respectively) said that they used alternative means of communication because of the face masks. The eye area and eye movements, the tone of voice and the reaction of the forehead or eyebrows were mentioned most frequently.

Concerning the interaction and relationship formation, the patients particularly stated in the free text fields that they have difficulties understanding and expressing humorous remarks and irony and that misinterpretations and misunderstandings tend to occur between the patients rather than with the staff. However, the face mask also helps them to differentiate their own feelings and needs from other patients and healthcare professionals. Nevertheless, it is more problematic to perceive emotions and the wellbeing of other patients in order to then help other patients. The patients stated that the face masks inhibit personal (physical) contact with fellow patients and staff. Many patients criticized the lack of a “handshake” or “consolatory touch” on the shoulder by the professional team.

Several patients stated a feeling of “anonymization” and “isolation,” which, however, would not be permanent. Many patients stated in the free text fields that the face masks as a “common evil” had strengthened the feeling of solidarity within the patient group. On the part of the Healthcare professionals, 85% denied a “we-feeling” created by the face masks. The healthcare professionals stated that the face masks changed the way they communicated with colleagues (*n* = 16; 61.6%). They were more likely to speak in a consciously friendly, clear, and direct way (42%). As a result of the face masks and distance regulations, there was less exchange about hospital structures (*n* = 23; 85.2%) and less interchange about patients (*n* = 15; 55.6%). Over 65% found themselves less attached to colleagues and 45% found themselves alienated by the face masks. Moreover, many of them stated in the free text fields that the consultation situation, the arrangement of breaks and therapy planning were also negatively changed by this feeling of alienation.

## Discussion

The aim of this study was to assess the multifaceted impact of face masks during the challenging circumstances of the corona pandemic on both ends: patients and health professionals. The majority of patients and healthcare professionals somehow reported an impact of the masks on psychotherapy, however, healthcare professionals had greater concerns about masked therapy than patients. Also, perceptions strongly differed for different types of therapy: healthcare professionals saw the greatest negative influence of face masks for interactional group therapy, body therapy, music and art therapy. Patients reported an especially negative impact during relaxation and stabilization techniques, music and art therapy followed by individual guidance and therapeutic counseling by nurses. One possible explanation for the diverging results might be that patients have different expectations of non-verbal therapies such as music, art, and body-oriented therapies (Junne and Zipfel, 2016). Since communication in non-verbal therapies is of secondary importance, patients may find it more difficult to fully engage in therapy despite the mask. As wearing of masks requires the use of other communication channels, over 85% of respondents reported using alternative means of communication given the face masks which mostly included the eye area and eye movements, tone of voice, and forehead or eyebrow responses.

On health professional side, masks lead to louder, more clearly and kindly communication. In line with this, Hüfner et al. (2020) conclude that raising emotional awareness in patients with mental disorders (and perhaps in the healthcare professionals themselves) occurs by addressing the “masked emotions” directly and explicitly. Nevertheless, the majority of healthcare professionals stated that the mask did not have a great impact on the content of their sessions as that they do not consciously work in a more resource-oriented or less confrontational or exposed way, except for particularly sensitive patient groups and in conflict situations. In line with this, Barrick et al. (2021) found an increase in the usage of facial visual features with increasing mask exposure. The more people interacted with other mask wearers, the more they learned to focus on visual cues from the eye area of the face, which can also be transferred to clinical interactions in the hospital.

The greatest difference concerning the negative influence of face masks was found within individual therapy on both ends, professionals and patients especially for the aspects of “being understood by the therapist,” “my therapist means it honestly,” and “being able to trust the patient.” Although there are mixed findings on the assessment of the trustworthiness of a counterpart wearing a face mask, an overall tendency toward a negative bias in the assessment of trustworthiness can be derived from current literature (e.g., Carbon, 2020; Gabrieli and Esposito, 2021; Marini et al., 2022).

Our results find support by Biermann et al. (2021) who found a negatively biased perception of trustworthiness in faces covered with a face mask among healthy individuals. This link was amplified by the experience of high distress which also applies for highly strained healthcare professionals during the pandemic. Gabrieli and Esposito (2021) showed that, compared to a non-mask condition, age, and gender of the counterpart had an influence on the subjective perception of trust in masked interaction partners. Adults and older individuals and individuals of different gender were perceived to be less trustworthy when wearing a mask. These findings can be used to identify factors that influence the development of trust in psychotherapy and, if possible, to take them into account in the selection of the therapist-patient dyad. Similarly, Cannito et al. (2022) found effects on decision-making patterns in interactions with masked, untrustworthy interaction partners.

Based on the evidence on the effect of covering the lower half of the face, the use of transparent face masks can be considered since they do not impair emotion recognition and trust attribution (Marini et al., 2021). Especially for structurally impaired patients and for patients suffering from disorders who are known to have a negative bias on facial recognitions such as borderline personality disorder (Domes et al., 2009) or schizophrenia (Ventura et al., 2013) transparent masks might serve as a useful tool.

Other striking differences were found concerning the social sense of belonging between and also within the groups. On the one hand, patients perceived the mask as a sign of “feeling connected,” or as a “sorrow shared.” Also 85% of patients said that the face mask did not discourage them from approaching staff (or fellow patients) or asking for help. On the other hand, concerning interactional group therapy, the patients stated that due to the face masks there is a barrier in “showing feelings” in the group. The patients also particularly expressed that they had difficulties in responding to ambiguous remarks or in adequately perceiving requests for help from other patients. Indeed, these observations align well with the existing literature, which shows that the reading and interpretation of emotions can be “severely” disturbed by the presence of a mask, and that confusion and misinterpretation of certain emotions can occur (Carbon, 2020; Lau, 2021; Grahlow et al., 2022). Also, these results are in line with general findings on impaired social interaction skills (for instance shaping and initiating social interactions, impairments concerning theory of mind) that can be symptom and cause of psychiatric diseases (Schilbach et al., 2013). On the positive side, this new “shared experience” of social interaction and finding alternative ways to get in touch with each other may have a positive and community-building effect. However, practitioners reported a sense of alienation and anonymization from the team. Current literature suggests that face masks not only reduce the ability to accurately categorize emotional expressions, they also make the other person seem less “close,” less trustworthy, likeable, and intimate (Biermann et al., 2021; Grundmann et al., 2021). It can be assumed that the practitioner’s perception was also due to an actual enforced individualization since otherwise usual group meetings and common meals were no longer possible during the pandemic.

Concerning feasibility, both sides raised concerns and complaints wearing the mask yet on the patients side, concerns were more accentuated and related to mental health problems, such as increased feeling of fear or panic. On the practitioner side, it was pointed out that climbing stairs was impaired wearing a mask. Steinhilber et al. (2022) investigated whether wearing a medical face mask (MedMask) affects the physical ability to work. Yet, they found that wearing face masks for infection prevention measures during the COVID-19 pandemic does not lead to any relevant additional physical demands, although a slightly higher breathing effort is required. It can be assumed that the findings were attributable to the time of the study and the fact that the familiarization effect of wearing a mask has not yet occurred.

During this study, the team of healthcare professionals was entrusted with daily changing regularities (testing, taking fevers, keeping distances, hygiene measures, isolation, etc.) in addition to providing medically and therapeutically care to their patients. Possibly, the assessments of the influence of the face masks in psychotherapy are also affected by these aspects above. Most patients stated in the last free text field on the questionnaire that they were very grateful that further psychotherapeutic counseling could be offered despite the current pandemic. This form of gratefulness could also influence the rather marginal-negative assessment of the patients.

To the best of our knowledge, this is the first study that examined bilateral therapeutic experiences in an in-patient setting during the early phases of the pandemic between July and October 2020. Hospitals represent highly complex workplaces characterized by high demands and low levels of control anyway. Work-related stress, reduced wellbeing, burnout and symptoms of mental illness such as depression are prevalent among healthcare professionals (e.g., Schulz et al., 2009; Klein et al., 2011; Mulfinger et al., 2020). Healthcare professionals often manifest attitudes and behaviors that are characterized by a high level of commitment and self-overload, with little ability to distance themselves from professional problems. Since the beginning of 2020, hospital employees have been additionally burdened by the acute health crisis due to the COVID-19 pandemic (Sanghera et al., 2020; Vindegaard and Benros, 2020). Direct contact with patients, quarantine experiences, and perceived health risks were identified as risk factors for increased stress (e.g., Mulfinger et al., 2020).

This report presents the subjective results of the survey of patients’ and healthcare professionals’ self and peer assessments, which are always susceptible to bias. We conducted the study during a period when there is little chance of habituation effects from mask wearing. Most patients had previous psychotherapy experience without a mask. Nevertheless, not conducting a randomized controlled trial, we cannot make causal statements about the quantitative and qualitative effects of face masks on the three dimensions of (i) feasibility, (ii) psychotherapeutic treatment, and (iii) communication, emotion, and relationships. The rapidly changing situational factors and the inter- and intrapsychic reactions toward those factors will possibly make the associations found here appear different when the survey is repeated at a later point in time. A direct influence of masked psychotherapy on the attainment of individual therapy objectives, conflict -and symptom management, and relationship skills cannot be assessed with this study either. The response rate for this survey was high with over 95% (for patients) and over 86% (for health professionals), but we refer to our sample size of *N* = 83 participants as a possible limitation.

The results from this study indicate that the face mask leads to more negative assessments on the part of the healthcare professionals than on the part of the patients. The majority of the patients evaluate their psychotherapy as very profitable in spite of the mask. In individual therapy, the mask seems to have a rather marginal influence on psychotherapy and the relationship with the psychotherapist as seen by the patients. Most patients stated that they used alternative cues. In interactional group therapy, the effects of the mask were interfering. Patients should be informed about the possible influences of the face mask on the recognition of emotions, possible misinterpretations and possibilities of compensation through alternative stimuli (e.g., area of the eyes) and encouraged to ask for information. Especially in group therapy, with patients from other cultural backgrounds and when assistance is needed (e.g., impaired hearing) or in cases of profound mental illness, non-verbal gestures, and body language should be matched to the intended emotional expression. Mitzkovitz et al. (2022) have compiled suggestions for conducting masked psychotherapy in their review article.

## Data availability statement

The raw data supporting the conclusions of this article will be made available by the authors, without undue reservation.

## Ethics statement

The responsible Ethics Committee of the University Hospital and Medical Faculty of the University of Tübingen was informed and reviewed the project (project number 685/2002A). For completely anonymous data, consultation and approval on the collection, analysis and publication by the ethics committee is not required. Written informed consent for participation was not required for this study in accordance with the national legislation and the institutional requirements.

## Author contributions

RE was mainly responsible for the conception and design of the study and wrote the first draft of the manuscript. KEG, AH-W, SZ, and FJ gave substantial input. CW acquired the data. RE, SHA, and CW analyzed and interpreted the data with substantial input from HW, TF-W, and NM. RE and SHA drafted the table and figures. RE, SHA, and KEG drafted the revision of the manuscript. All authors commented and revealed the final manuscript.
